# Identifying Effective Antiviral Drugs Against SARS-CoV-2 by Drug Repositioning Through Virus-Drug Association Prediction

**DOI:** 10.3389/fgene.2020.577387

**Published:** 2020-09-16

**Authors:** Lihong Peng, Xiongfei Tian, Ling Shen, Ming Kuang, Tianbao Li, Geng Tian, Jialiang Yang, Liqian Zhou

**Affiliations:** ^1^School of Computer Science, Hunan University of Technology, Zhuzhou, China; ^2^Geneis (Beijing) Co., Ltd., Beijing, China

**Keywords:** SARS-CoV-2, antiviral drugs, drug repositioning, virus-drug association, regularized least square, bipartite local model, neighbor association information

## Abstract

A new coronavirus called SARS-CoV-2 is rapidly spreading around the world. Over 16,558,289 infected cases with 656,093 deaths have been reported by July 29th, 2020, and it is urgent to identify effective antiviral treatment. In this study, potential antiviral drugs against SARS-CoV-2 were identified by drug repositioning through Virus-Drug Association (VDA) prediction. 96 VDAs between 11 types of viruses similar to SARS-CoV-2 and 78 small molecular drugs were extracted and a novel VDA identification model (VDA-RLSBN) was developed to find potential VDAs related to SARS-CoV-2. The model integrated the complete genome sequences of the viruses, the chemical structures of drugs, a regularized least squared classifier (RLS), a bipartite local model, and the neighbor association information. Compared with five state-of-the-art association prediction methods, VDA-RLSBN obtained the best AUC of 0.9085 and AUPR of 0.6630. Ribavirin was predicted to be the best small molecular drug, with a higher molecular binding energy of −6.39 kcal/mol with human angiotensin-converting enzyme 2 (ACE2), followed by remdesivir (−7.4 kcal/mol), mycophenolic acid (−5.35 kcal/mol), and chloroquine (−6.29 kcal/mol). Ribavirin, remdesivir, and chloroquine have been under clinical trials or supported by recent works. In addition, for the first time, our results suggested several antiviral drugs, such as FK506, with molecular binding energies of −11.06 and −10.1 kcal/mol with ACE2 and the spike protein, respectively, could be potentially used to prevent SARS-CoV-2 and remains to further validation. Drug repositioning through virus–drug association prediction can effectively find potential antiviral drugs against SARS-CoV-2.

## Introduction

Last December 2019, a novel coronavirus called SARS-CoV-2 by the World Health Organization (WHO), first found in Wuhan, China, was rapidly spreading around the world (Kaiser et al., 2020; Sanche et al., 2020). The SARS-CoV-2 outbreak was declared as a global public health emergency by WHO, and a total of 16,558,289 cases have been confirmed with another 656,093 deaths throughout the world by July 29th, 2020 (World Health Organization [WHO], 2020). SARS-CoV-2 caused a severe acute respiratory syndrome named COVID-19, and no special vaccine or antiviral drug against SARS-CoV-2 has been found at present (Lu, 2020; Wang et al., 2020c). Therefore, finding a special antiviral drug as soon as possible is urgent to stop the spread of SARS-CoV-2 (Lu, 2020; Zhang et al., 2020a).

However, designing a new drug to treat COVID-19 in a short time is almost impossible (Zhang et al., 2020a). One of the best strategies is drug repositioning (Chen et al., 2012, 2016; Peng et al., 2017a; Beck et al., 2020). By repositioning already commercialized drugs, the undesired effects can be inferred to find new uses for these drugs. This strategy can thus greatly shorten the time required for an antiviral drug against SARS-CoV-2.

Although little is known about SARS-CoV-2, its complete genome sequence is strongly homologous to SARS-CoV (Huang et al., 2020; Morse et al., 2020). Therefore, in this study, to prioritize available FDA-approved antiviral drugs against SARS-COV-2 for further clinical trials, 11 well-studied viruses similar to SARS-CoV-2 were selected and 96 virus–drug associations (VDAs) with these 11 viruses were integrated. Regularized least squared classifier (RLS), bipartite local model (BLM), and neighbor association information were applied in our new algorithm named VDA-RLSBN to find novel VDAs for new virus (especially for SARS-CoV-2) or new drug. The results showed that ribavirin, remdesivir, and chloroquine may be antiviral drugs against SARS-CoV-2.

Molecular docking techniques investigate the behavior of small molecular drugs in the binding site of a target protein. As more target protein structures are confirmed experimentally, molecular docking approaches are widely applied to drug design (Zhang et al., 2020b). AutoDock (Goodsell et al., 1996; Ruyck et al., 2016) is an available software applied to identify the bound conformations of a small molecular drug to a macromolecular target. The AutoDock affinity scoring function is applied to rank the candidate poses based on the sum of the van der Waals and electrostatic energies. We conducted molecular docking between the predicted top 10 antiviral drugs against SARS-CoV-2 and two target proteins including the spike protein of SARS-CoV-2 and human angiotensin-converting enzyme 2 (ACE2) molecule (Wang et al., 2020a). The molecular binding energies between the above three drugs and ACE2 are ribavirin with −6.39 kcal/mol, remdesivir with −7.4 kcal/mol, and chloroquine with −6.29 kcal/mol. These three small molecules have been under clinical trial or supported by recent publications. In addition, we found that FK506 shows higher molecular binding energies of −10.1 kcal/mol and −11.06 kcal/mol with these two targets, which suggest that FK506 may be applied to stop COVID-19 although there is no report about its association with SARS-CoV-2.

## Materials and Methods

### Dataset

Aiming at identifying potential VDAs related to SARS-CoV-2, 96 known VDAs between 11 viruses similar to SARS-CoV-2 and 78 small molecular drugs were selected from the DrugBank (Wishart et al., 2018), NCBI (Sayers et al., 2020), and PubMed (Canese and Sarah, 2013) databases. The element ${\text{y}}_{i\,j}^{o\,r\,i}$ in the VDA matrix *Y*^ori^ ∈ ℜ^n×m^ was represented as

(1) $${\text{y}}_{i\,j}^{o\,r\,i}=\left\{ \begin{array}{cc} 1 & \mathrm{if}\,\mathrm{the}\,i\,\mathrm{th}\,\mathrm{virus}\,\mathrm{associates}\,\mathrm{with}\,\mathrm{the}\,j\,\mathrm{th}\,\mathrm{drug}\\ 0 & \text{otherwise} \end{array}\right.$$

These similar viruses included SARS-CoV (Ding et al., 2004), MERS-CoV (Groot et al., 2013), human immunodeficiency virus type 1 (Wei et al., 1995) and type 2 (Guyader et al., 1987) (HIV-1 and HIV-2), chronic hepatitis C virus (HCV) (Jacobson et al., 2011), influenza A viruses [A-H1N1 (Kumar et al., 2009), A-H5N1 (Subbarao et al., 1998), A-H7N9 (Gao et al., 2013)], Hendra virus (Bonaparte et al., 2005), human cytomegalovirus (Cobbs et al., 2002), and respiratory syncytial virus (Hall, 2001). Complete genome sequences of these 11 viruses and SARS-CoV-2 were downloaded from the NCBI database, and virus similarity matrix *S*_*v*_ ∈ ℜ^n×n^ was computed based on MAFFT, a multiple-sequence alignment software. Chemical structures of drugs were downloaded from the DrugBank database, and drug similarity matrix *S*_*d*_ ∈ ℜ^m×m^ was obtained by RDKit, an open-source cheminformatics tool. The details are shown in Table 1.

**TABLE 1:** Statistics of viruses and drugs.

Virus	No. of drugs	Virus	No. of drugs
SARS-CoV	15	Hendra virus	1
MERS-CoV	9	HIV-1	35
A-H1N1	4	HIV-2	3
A-H5N9	2	HCV	15
A-H7N9	4	Respiratory syncytial virus	2
Human cytomegalovirus	6	SARS-CoV-2	0

### Methods

#### Problem Formalization

Bleakley and Yamanishi (2009) represented a drug–target interaction network as a bipartite graph and developed a BLM-based method to predict possible drug–target interactions. The proposed method first inferred targets of a given FDA-approved drug and drugs targeting a known protein and then combined these two independent predictions. The results demonstrated the excellent performance of BLM. Similar to the drug–target interaction network, the VDA network can also be taken as a bipartite graph. Results in this study are thus presented to evaluate the prediction performance in each of the following four cases for a given putative virus–drug pair:

• The virus with at least one known drug and the drug with at least one known virus.
• The virus with at least one known drug and the drug without any known virus (new drug).
• The virus without any known drug (new virus) and the drug with at least one known virus.
• New virus and new drug.

**FIGURE 1:**
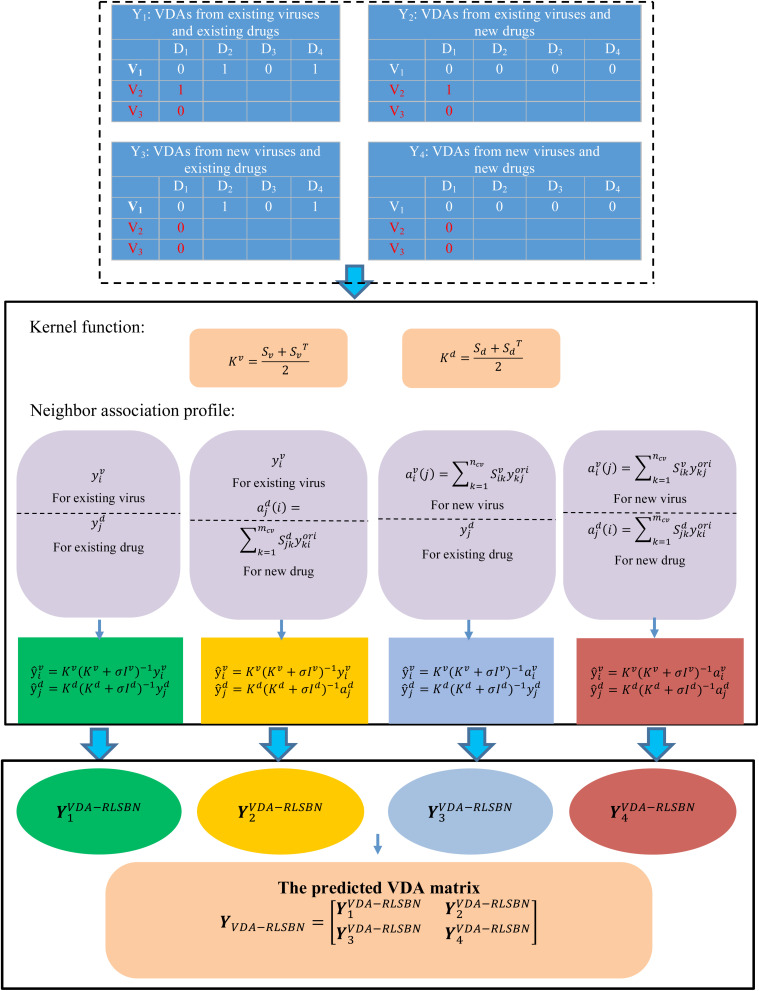
Flowchart of VDA-RLSBN.

Based on these four cases, we represent a VDA network as a bipartite graph and thus the predicted VDA matrix $Y_{n\times m}^{\text{pre}}$ can be denoted as Eq. (2):

(2) $$Y_{n\times m}^{\text{pre}}=\left[\begin{array}{c} {\left(Y_{1}\right)}_{n_{c\,v}\times m_{c\,v}}\\ {\left(Y_{3}\right)}_{\bar{n}\times m_{c\,v}} \end{array}\,\begin{array}{c} {\left(Y_{2}\right)}_{n_{c\,v}\times \bar{m}}\\ {\left(Y_{4}\right)}_{\bar{n}\times \bar{m}} \end{array}\right]$$

where $\bar{n}=n-n_{c\,v}$ is the number of new viruses (for example, SARS-CoV-2), and $\bar{m}=m-m_{c\,v}$ is the number of new drugs. *Y*_1_ represents VDAs from *n*_*cv*_ existing viruses and *m*_*cv*_ existing drugs, *Y*_2_ represents VDAs from *n*_*cv*_ existing viruses and $\bar{m}$ new drugs, *Y*_3_ denotes VDAs from $\bar{n}$ new viruses and *m*_*cv*_ existing drugs, and *Y*_4_ denotes VDAs from $\bar{n}$ new viruses and $\bar{m}$ new drugs. Our aims are to identify potential VDAs in the subnetwork *Y*_1_ as well as in *Y*_2_, *Y*_3_, and *Y*_4_. Figure 1 shows the flowchart of VDA-RLSBN.

#### Regularized Least Square

To infer possible VDA candidates, we develop an RLS-based VDA identification model (VDA-RLS) to compute the association profile $\hat{y}$ for each virus–drug pair:

(3) $$\hat{y}=K\,{\left(K+\sigma \,I\right)}^{-1}\,y$$

where *K* represents the kernel matrix, *y* denotes the original association profile, and σ is a regularization parameter.

To compute VDA matrix *Y*_1_ from *n*_*cv*_ existing viruses and *m*_*cv*_ existing drugs, we consider the ensemble of independent virus-based prediction and drug-based prediction with RLS. The solution of *Y*_1_ can be thus divided down into the following four steps:

Step 1 For a given virus *v*_*i*_ with at least one known association, its new association profile ${\hat{y}}_{i}^{v}$ can be computed from its original association profile $y_{i}^{v}$ and the kernel matrix *K*_*v*_ based on RLS classifier:

(4) $${\hat{y}}_{i}^{v}=K_{v}\,{\left(K_{v}+\sigma \,I_{v}\right)}^{-1}\,y_{i}^{v}$$

where $K_{v}=\left(S_{v}+S_{v}^{T}\right)/2$, and $y_{i}^{v}$ represents the *i*th row of *Y*^ori^. We can compute virus-based VDA matrix *Y*^*v*^ by Eq. (4).

Step 2 For a given drug *d*_*j*_ with at least one known association, its new association profile ${\hat{y}}_{j}^{d}$ can be computed from its original association profile $y_{j}^{d}$ and the kernel matrix *K*_*d*_ based on RLS classifier:

(5) $${\hat{y}}_{j}^{d}=K_{d}\,{\left(K_{d}+\sigma \,I_{d}\right)}^{-1}\,y_{j}^{d}$$

where $K_{d}=\left(S_{d}+S_{d}^{T}\right)/2$, and $y_{j}^{d}$ represents the *j*th column of *Y*^ori^. We can compute drug-based VDA matrix *Y*^*d*^ by Eq. (5).

Step 3 Integrate *Y*^*v*^ with the element $y_{i\,j}^{v}$ and *Y*^*d*^ with the element $y_{i\,j}^{d}$ to compute the predicted VDA matrix *Y*^RLS^ based on RLS:

(6) $$y_{i\,j}^{\text{RLS}}=\max\left(y_{i\,j}^{v},y_{i\,j}^{d}\right)$$

Step 4 Obtain *Y*_1_ by Eq. (7):

(7) $$Y_{1}=Y^{o\,r\,i}+Y^{R\,L\,S}$$

#### Regularized Least Square With Neighbor Association Information

We can identify novel VDAs between existing viruses and existing drugs, or known/new viruses and new/existing drugs based on RLS and BLM. However, VDA-RLS was not able to predict associations between new viruses and new drugs. To solve this problem, we developed a VDA prediction model (VDA-RLSBN) by integrating neighbor association information into the RLS model.

Based on the “guilt-by-association” method, similar viruses/drugs tend to associate with similar drugs/viruses, so the association profile of an unknown virus could be possibly found by its neighbors’ association information. Viruses highly similar to a new virus can be considered as its neighbors. Since the new virus has no associated drugs (i.e., its current association profile is a vector with all the elements of 0), complete genome sequence similarity of viruses is applied to define its neighbors.

For a new virus *v*_*i*_, its association weight with a drug *d*_*j*_ can be computed by its neighbors’ associations with *d*_*j*_ and its association profile $a_{i}^{v}\,\left(j\right)$ is defined as Eq. (8):

(8) $$a_{i}^{v}\,\left(j\right)=\sum_{k=1}^{n_{c\,v}}S_{i\,k}^{v}\,y_{k\,j}^{o\,r\,i}$$

where $S_{i\,k}^{v}$ is the complete genome sequence similarity between two viruses *v*_*i*_ and *v*_*j*_. $a_{i}^{v}\,\left(j\right)>0$ when the *j*th associated drug *d*_*j*_ exists, i.e., $y_{k\,j}^{\text{ori}}>0$ for at least one *k* and $a_{i}^{v}\,\left(j\right)=0$ when the *j*th associated drug *d*_*j*_ is new, i.e., $y_{k\,j}^{\text{ori}}=0$ for all *k*. $a_{i}^{v}\,\left(j\right)$ is normalized to make its value in the range of [0,1] by Eq. (9):

(9) $$a_{i}^{v}\,\left(j\right)=\frac{\left(a_{i}^{v}\,\left(j\right)-{\text{min}}_{k}\,a_{i}^{v}\,\left(k\right)\right)}{\left({\text{max}}_{k}\,a_{i}^{v}\,\left(k\right)-{\text{min}}_{k}\,a_{i}^{v}\,\left(k\right)\right)}$$

Also, an independent virus-based association profile $y_{i}^{v}$ for a virus–drug pair can be represented as Eq. (10):

(10) $${\hat{y}}_{i}^{v}=K_{v}\,{\left(K_{v}+\sigma \,I_{v}\right)}^{-1}\,a_{i}^{v}$$

Similarly, for a new drug *d*_*j*_, its association profile $y_{j}^{d}$ for the same virus–drug pair can be represented as Eq. (10):

(11) $${\hat{y}}_{j}^{d}=K_{d}\,{\left(K_{d}+\sigma \,I_{d}\right)}^{-1}\,a_{j}^{d}$$

where $a_{j}^{d}$ denotes the neighbor association profile of *d*_*j*_.

The final VDA network can be represented as

(12) $$Y_{\text{VDA}-\text{RLSBN}}=\left[\begin{array}{c} Y_{1}^{\text{VDA}-\text{RLSBN}}\\ Y_{3}^{\text{VDA}-\text{RLSBN}} \end{array}\;\begin{array}{c} Y_{2}^{\text{VDA}-\text{RLSBN}}\\ Y_{4}^{\text{VDA}-\text{RLSBN}} \end{array}\right]$$

where $Y_{1}^{\text{VDA}-\text{RLSBN}}$ can be computed by Eqs (4–7); $Y_{2}^{\text{VDA}-\text{RLSBN}}$ can be computed by Eqs (4), (11), and (6); $Y_{3}^{\text{VDA}-\text{RLSBN}}$ can be obtained by Eqs (10), (5), and (6); and $Y_{4}^{\text{VDA}-\text{RLSBN}}$ can be obtained by Eqs (10), (11), and (6). Specially, the VDA matrix related to SARS-CoV-2 can be obtained from $Y_{3}^{\text{VDA}-\text{RLSBN}}$.

Finally, we used AutoDock to analyze the druggability of the predicted top 10 chemical agents and their binding activities with two target proteins including the SARS-CoV-2 spike protein and ACE2.

## Results

### Evaluation Metrics and Experimental Settings

In this section, we performed extensive experiments to evaluate our proposed VDA-RLSBN method. We compared VDA-RLSBN with five state-of-the-art machine learning-based models, including LRLSHMDA (Wang et al., 2017), SMiR-NBI (Li et al., 2016), CMF (Zheng et al., 2013), NetLapRLS (Xia et al., 2010), and WNN-GIP (Laarhoven and Marchiori, 2013). The experiments were performed on a MAC with 2.4 GHz Inter Core i5, 8 GB 2133 MHz LPDDR3 of the RAM and OS Catalina 10.15.4 operating system.

Sensitivity, specificity, accuracy, AUC, and AUPR are widely applied to evaluate various machine learning-based models. In this study, we used these five metrics to measure the performance of five state-of-the-art models and VDA-RLSBN. Accuracy denotes the ratio of correctly predicted VDAs to all VDAs. Sensitivity denotes the ratio of correctly predicted positive VDAs to all positive VDAs. Specificity is the ratio of correctly predicted negative VDAs to all negative VDAs. AUC is the area under the ROC curve. The ROC curve can be plotted by a true positive rate [TPR, i.e., Eq. (13)] and a false-positive rate [FPR, i.e., Eq. (14)].

(13) TPR=T⁢P/(T⁢P+F⁢N)

(14) FPR=F⁢P/(F⁢P+T⁢N)

where TPR represents the ratio of correctly predicted positive VDAs to all positive VDAs and FPR represents the ratio of mistakenly predicted positive VDAs to all negative VDAs.

AUPR is the area under the PR curve. The PR curve can be plotted by precision and recall. Precision represents the ratio of correctly predicted positive VDAs to all predicted positive VDAs, and recall represents the ratio of correctly predicted positive VDAs to all positive VDAs.

(15) $$\text{Precision}=\frac{T\,P}{T\,P+F\,P}$$

(16) $$\text{Recall}=\frac{T\,P}{T\,P+F\,N}$$

where *TP*,*FP*,*TN*,and*FN* represent true positive, false positive, true negative, and false negative, respectively. Generally, larger AUC/AUPR value denotes better performances.

We used five-fold cross validation to train our proposed VDA-RLSBN method. In each round, 80% of VDAs in the known VDA network was used as a training set and the remaining 20% of VDAs was the test set. The experiments were performed 100 times, and the final performance was on average over 100 times. In each round, a virus/drug is new if all of its associated drugs/viruses are selected as a test set.

For the parameters in five comparative methods and VDA-RLSBN, we conducted grid search to determine their optimal values. In VDA-RLSBN, we set the parameter σ in the range of [0, 0.1, 0.2, …, 1] and found that VDA-RLSBN obtained the best performance when σ is set as 0.4. In LRLSHMDA, we set the parameter *lw* in the range of [0, 0.1, 0.2, …, 1] and found that LRLSHMDA obtained better accuracy when *lw* is set as 0.1. In CMF, we set the parameters λ_*l*_,λ_*d*_,andλ_*t*_ in the range of [2^−2^,…,  2^1^], [2^−3^,…, 2^5^], and [2^−3^,…,  2^5^], respectively. We found that CMF obtained better performance when λ_*l*_ = 1, λ_*d*_ = 0.25, and λ_*t*_ = 0.125. In NetLapRLS, we set four parameters γ_*d*_, γ_*t*_, β_*d*_, and β_*t*_ in the range of [1*e*^−6^,…, 1*e*^2^] and found that NetLapRLS performed better when these four parameters were set as *1e-6*. In WNN-GIP, we set five parameters *T*, α_*d*_, α_*t*_, σ, and γ in the range of [0, 0.1,…, 1.0] and found that WNN-GIP obtained the optimal performance when *T* = 0.7, α_*d*_ = 0.6, α_*t*_ = 0.6, σ = 1, and γ = 0.5. All parameters in these six models were set as the corresponding values where the corresponding method obtained the optimal performance.

### Comparison With Five State-of-the-Art Methods

The performance of our proposed VDA-RLSBN and these five machine learning-based models is shown in Table 2. The best performance in each row is shown in bold in Table 2. LRLSHMDA (Wang et al., 2017), NetLapRLS (Xia et al., 2010), WNN-GIP (Laarhoven and Marchiori, 2013), and VDA-RLSBN are RLS-based methods. LRLSHMDA (Wang et al., 2017) used Laplacian RLS to tackle microbe–disease association prediction, NetLapRLS (Xia et al., 2010) extended the standard Laplacian RLS incorporating drug–target network, and WNN-GIP (Laarhoven and Marchiori, 2013) integrated a simple weighted nearest neighbor method and Gaussian kernels into RLS. SMiR-NBI (Li et al., 2016) constructed a heterogeneous network connecting genes, drugs, and miRNAs and then combined a network-based inference algorithm to characterize the responses of anticancer drugs. CMF (Zheng et al., 2013) was a collaborative matrix factorization-based drug–target interaction prediction method.

**TABLE 2:** The performance of VDA-RLSBN with other five methods.

Methods	Accuracy	Sensitivity	Specificity	AUC	AUPR
LRLSHMDA	0.5841	0.6702	0.5823	0.8303	0.1778
SMiR-NBI	0.2080	0.8437	0.1935	0.5721	0.4912
CMF	0.8980	0.8971	0.9916	0.7500	0.4210
NetLapRLS	0.8974	0.8974	**0.9992**	0.6758	0.1777
WNN-GIP	0.8786	0.8961	0.9072	0.8491	0.5356
VDA-RLSBN	**0.9298**	**0.9279**	0.9841	**0.9085**	**0.6630**

The results showed that VDA-RLSBN outperformed LRLSHMDA, SMiR-NBI, CMF, and WNN-GIP in terms of five evaluation metrics. Although the specificity value of VDA-RLSBN is slightly lower compared to NetLapRLS, its AUC and AUPR are significantly higher than NetLapRLS. Since AUC and AUPR are more important evaluation metrics compared to other three measurements, VDA-RLSBN, with the highest AUC and AUPR, is considered to be better in finding potential VDAs of novel viruses.

Among six VDA prediction methods, LRLSHMDA, NetLapRLS, WNN-GIP, and VDA-RLSBN are RLS-based methods. VDA-RLSBN obtained better performance than the other three methods. Although other RLS-based prediction methods have good performance, they cannot predict the relationship between new drug candidates and new candidate targets. If a virus/drug has no known drug/virus, it is a new virus/drug. Since there are many new viruses/drugs, our proposed VDA-RLSBN approach learned labeled information from neighbors and used the information to train the model and make predictions. So VDA-RLSBN obtained better performance compared to other RLS-based methods. The results suggest that RLS combining neighbor association information can better identify new VDAs.

### Case Study

The prediction performance of the proposed VDA-RLSBN method was confirmed in the last section. As a means to finding potential antiviral drugs against SARS-CoV-2, small molecular drugs were ranked based on the association scores with SARS-CoV-2 and the top 10 drugs with the highest scores were listed in Table 3. Among the predicted top 10 VDAs, 4 VDAs are reported by related literature, that is, 40% small molecular drugs are confirmed to be possible antiviral drugs against SARS-CoV-2.

**TABLE 3:** The predicted top 10 drugs associated with SARS-CoV-2.

Rank	Drug	Confirmed
1	Ribavirin	10.1038/d41573-020-00016-0 PMID:32034637
2	Remdesivir	PMID:32036774, 32035533, 32035018, 31971553, 32022370, 31996494, 32020029 10.1101/2020.01.28.922922 10.26434/chemrxiv.11831101.v1
3	Mycophenolic acid	Unconfirmed
4	Chloroquine	PMID:32020029
5	Phenothiazine	Unconfirmed
6	Mizoribine	10.20944/preprints202002.0061.v1
7	FK506	Unconfirmed
8	Pentoxifylline	Unconfirmed
9	6-Azauridine	Unconfirmed
10	Protein phosphatase 1	Unconfirmed

Ribavirin is inferred to be the best small molecular drug against SARS-CoV-2. It is a broad-spectrum antiviral drug that can inhibit the replication of respiratory syncytial virus (Laarhoven and Marchiori, 2013). For example, it has been applied to prevent respiratory syncytial virus infection in lung transplant recipients (Hayden and Whitley, 2020) and specially used to treat SARS-CoV and MERS-CoV (Permpalung et al., 2019). Similar to SARS-CoV and MERS-CoV, SARS-CoV-2 is a respiratory syndrome betacoronavirus and may cause serious respiratory diseases. A few studies (Li and De, 2020; Wang et al., 2020b) have reported that ribavirin may take an inhibitory effect on SARS-CoV-2. More importantly, remdesivir and chloroquine are inferred to be other effective antiviral drugs. Wang et al. (2020b) presented that remdesivir and chloroquine can effectively inhibit SARS-CoV-2 and they have been used in the clinical stage. These results suggest that ribavirin, remdesivir, and chloroquine may be applied to the treatment of COVID-19.

### Molecular Docking

We conducted molecular docking between the predicted top 10 small molecules and the SARS-CoV-2 spike protein/ACE2. The chemical structures of these small molecular drugs were downloaded from the DrugBank database. The structure of the virus spike protein was obtained based on homologous modeling from Zhang Lab (2020). The structure of ACE2 can be downloaded from the RCSB Protein Data Bank (Helen et al., 2000) (ID:6MJ0). AutoDock used the genetic algorithm as a search algorithm and selected the entire protein as a grid box.

The molecular binding energies between the predicted top 10 small molecules and these two target proteins are described in Table 4. The results show that the predicted top 10 drugs have higher molecular binding activities with the spike protein and/or ACE2. For example, ribavirin, which is predicted to be the most possible drug against SARS-CoV-2, has a higher molecular binding energy of −6.39 kcal/mol with ACE2. In addition, remdesivir, mycophenolic acid, and chloroquine are predicted to have higher association scores with SARS-CoV-2. These three small molecular drugs showed higher binding energies of −7.4, −5.35, and −6.29 kcal/mol with ACE2, respectively. More importantly, ribavirin, remdesivir, and chloroquine have been used for the treatment of SARS, which has about 79% sequence identity with SARS-CoV-2. So the potential use of these three small molecules as a treatment for COVID-19 may be under investigation. Interestingly, FK506 is an immunesuppressive drug and mainly used to decrease the activity of the immune system after organ transplant. The molecular docking results show that FK506 has a strong molecular binding energy of −11.06 and −10.1 kcal/mol with ACE2 and the spike protein, respectively, although it has a slightly lower rank in the predicted drugs against SARS-CoV-2 by VDA-RLSBN.

**TABLE 4:** The molecular binding energies between the predicted top 10 antiviral drugs and two target proteins.

Target protein	Drug	Binding energy
The spike protein	Ribavirin	–5.29
	Remdesivir	–5.22
	Mycophenolic acid	–3.6
	Chloroquine	–5.03
	Phenothiazine	–5.44
	Mizoribine	–6.07
	FK506	–10.1
	Pentoxifylline	–8.59
	6-Azauridine	–7.72
	Protein phosphatase 1	–8.46
ACE2	Ribavirin	–6.39
	Remdesivir	–7.4
	Mycophenolic acid	–5.35
	Chloroquine	–6.29
	Phenothiazine	–8.12
	Mizoribine	–7.62
	FK506	–11.06
	Pentoxifylline	–5.98
	6-Azauridine	–10.74
	Protein phosphatase 1	–9.13

**FIGURE 2:**
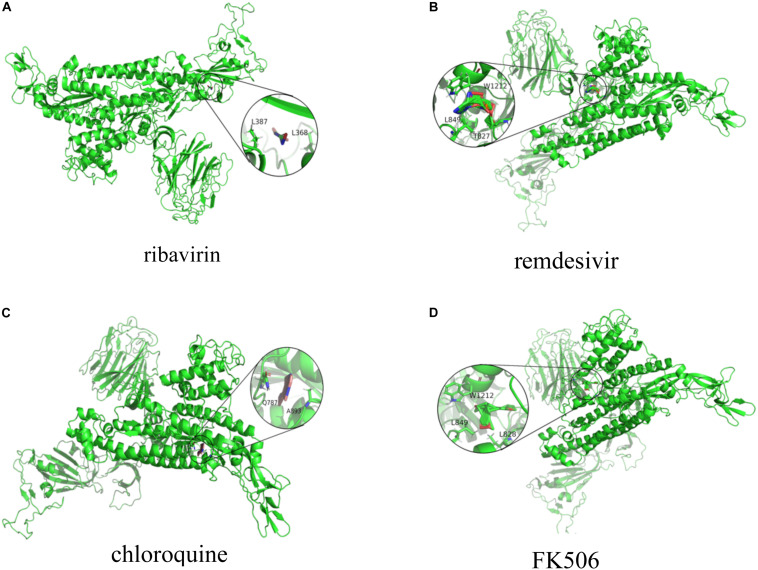
Molecular docking between **(A)** ribavirin, **(B)** remdesivir, **(C)** chloroquine, and **(D)** FK506 and the spike protein.

**FIGURE 3:**
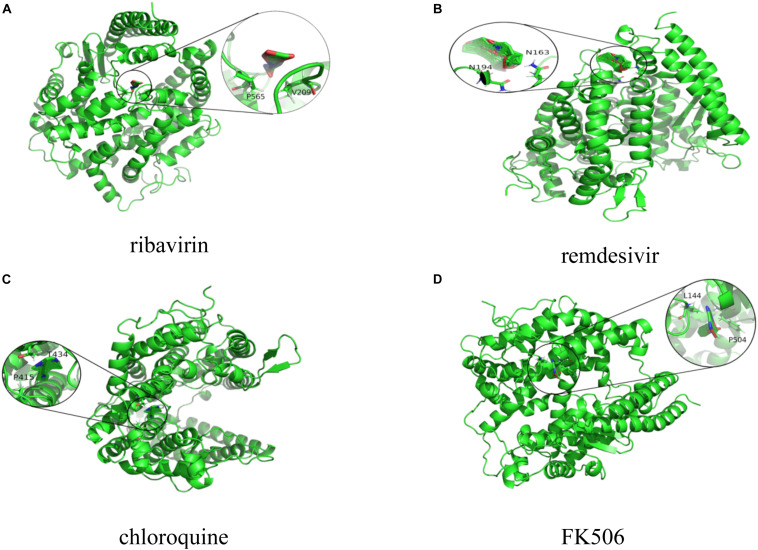
Molecular docking between **(A)** ribavirin, **(B)** remdesivir, **(C)** chloroquine, and **(D)** FK506 and ACE2.

Figures 2, 3 represent the docking results about four small molecules including ribavirin, remdesivir, chloroquine, and FK506 and two target proteins. The subfigure in each circle denotes the residues at the binding site of the SARS-CoV-2 spike protein/ACE2 and their corresponding orientations. For example, the amino acids L387, L368, P565, and V209 were inferred to be the key residues for ribavirin binding to the SARS-CoV-2 spike protein/ACE2 while L828, L849, W1212, N163, and N194 were inferred as the key residues for FK506 binding to the SARS-CoV-2 spike protein/ACE2.

## Discussion

With the spreading of SARS-CoV-2 around the world, the incidence rate is rapidly increasing, and lack of effective treatment options made it a public health threat. Therefore, various strategies are being exploited. Drug repositioning, aiming to offer a potentially valuable opportunity to find new clues of treatment for existing FDA-approved drugs, provides a far more rapid option to the clinic than novel drug design.

In the proposed VDA-RLSBN method, we predicted VDA candidates based on RLS and BLM. However, SARS-CoV-2 is a new coronavirus and has no associated drugs verified by biomedical experiments. We cannot find potential VDAs related to the virus by RLS and BLM. Therefore, we used association information of other RNA viruses similar to SARS-CoV-2 and similarities between SARS-CoV-2 and these viruses. The originality of our proposed method remains, predicting possible antiviral drugs against SARS-CoV-2 by drug repositioning through virus–drug association identification. More importantly, we integrated neighbor association information to RLS to find associated chemical agents for the new virus. The experimental results showed the merits of the VDA-RLSBN model. Higher AUC and AUPR indicated that the predicted antiviral drugs against SARS-CoV-2 are likely to be effective for preventing the rapid transmission of COVID-19.

VDA-RLSBN can obtain superior performance regardless of AUC, AUPR, accuracy, or sensitivity. This observation may be attributed to the following two features. First, VDA-RLSBN divides new VDA prediction into four cases based on BLM, a state-of-the-art method applied in various association prediction areas. More importantly, neighbor association information can help to identify possible antiviral drugs against new viruses (for example, SARS-CoV-2).

The proposed VDA-RLSBN approach is also helpful in designing and interpreting pharmacological experiments. The method can be further applied to select potential antiviral drugs against other new viruses, for example, infectious bronchitis virus.

## Conclusion

In this study, we considered the clues of treatment from SARS-CoV, MERS-CoV, and other diseases caused by single-strand RNA viruses and developed a VDA prediction method based on RLS, BLM, and neighbor association information. VDA-RLSBN inferred commercially available small molecular drugs that could be applied to experimental therapy options against SARS-CoV-2. We conducted molecular docking between the predicted four chemical compounds including ribavirin, remdesivir, chloroquine, and FK506 and two target proteins including the spike protein and ACE2. The results show that ribavirin, remdesivir, and chloroquine have better molecular binding activities with ACE2 and may be the best small molecular drugs against SARS-CoV-2. In addition, we found that several antiviral drugs, such as FK506, could be used to combat COVID-19. Nevertheless, the 4 predicted drugs ranked 1, 2, 4, and 6 have been supported by recent works. We hope that our predicted small molecules may be helpful in the prevention of the transmission of SARS-CoV-2.

In the future, we will develop ensemble frameworks (Hu et al., 2018; Peng et al., 2020) and positive-unlabeled learning methods (Lan et al., 2016a; Peng et al., 2017b) to further improve the prediction performance. More importantly, we will enlarge the existing dataset. We will also integrate various biological data including long noncoding RNA (Lan et al., 2017; Zhao et al., 2018; Liu et al., 2020) and disease symptom information (Lan et al., 2016b).

## Code Availability

Source code is freely downloadable at: https:// github.com/plhhnu/VDA-RLSBN/.

## Data Availability Statement

All datasets presented in this study are included in the article/Supplementary Material.

## Author Contributions

LP and XT contributed equally to this work. LP, XT, JY, and LZ designed the VDA-RLSBN method. XT and MK ran VDA-RLSBN. XT wrote the original manuscript. LP, TL, and JY revised the original draft. LS conducted molecular docking for the predicted results. LP, GT, JY, and LZ discussed the proposed method and gave further research. All authors read and approved the final manuscript.

## Conflict of Interest

GT, TL, and JY were employed by the company Geneis (Beijing) Co., Ltd. The remaining authors declare that the research was conducted in the absence of any commercial or financial relationships that could be construed as a potential conflict of interest.
